# Morphological and molecular characterization and phylogenetic relationships of a new species of trypanosome in *Tapirus terrestris* (lowland tapir), *Trypanosoma terrestris* sp. nov., from Atlantic Rainforest of southeastern Brazil

**DOI:** 10.1186/1756-3305-6-349

**Published:** 2013-12-11

**Authors:** Igor da Cunha Acosta, Andrea Pereira da Costa, Pablo Henrique Nunes, Maria Fernanda Naegeli Gondim, Andressa Gatti, João Luiz Rossi Jr, Solange Maria Gennari, Arlei Marcili

**Affiliations:** 1 Department of Preventive Veterinary Medicine and Animal Science, Faculty of Veterinary Medicine, University of São Paulo, São Paulo, SP Brazil; 2 Department of Biology, Biosciences Institute, UNESP, Rio Claro, SP Brazil; 3 Faculty of Veterinary Medicine, University of Vila Velha, Vila Velha, ES Brazil; 4 Marcos Daniel Institute, Vitória, ES Brazil

**Keywords:** *Trypanosoma*, Tapirs, Perissodactyla, Phylogeny, Atlantic rainforest, Taxonomy

## Abstract

**Background:**

The Lowland tapir (*Tapirus terrestris*) is the largest Brazilian mammal and despite being distributed in various Brazilian biomes, it is seriously endangered in the Atlantic Rainforest. These hosts were never evaluated for the presence of *Trypanosoma* parasites.

**Methods:**

The Lowland tapirs were captured in the Brazilian southeastern Atlantic Rainforest, Espírito Santo state. Trypanosomes were isolated by hemoculture, and the molecular phylogeny based on small subunit rDNA (SSU rDNA) and glycosomal-3-phosphate dehydrogenase (gGAPDH) gene sequences and the ultrastructural features seen via light microscopy and scanning and transmission electron microscopy are described.

**Results:**

Phylogenetic trees using combined SSU rDNA and gGAPDH data sets clustered the trypanosomes of Lowland tapirs, which were highly divergent from other trypanosome species. The phylogenetic position and morphological discontinuities, mainly in epimastigote culture forms, made it possible to classify the trypanosomes from Lowland tapirs as a separate species.

**Conclusions:**

The isolated trypanosomes from *Tapirus terrestris* are a new species, *Trypanosoma terrestris* sp. n., and were positioned in a new *Trypanosoma* clade, named *T. terrestris* clade.

**Electronic supplementary material:**

The online version of this article (doi:10.1186/1756-3305-6-349) contains supplementary material, which is available to authorized users.

## Background

*Trypanosoma* species are parasites of all vertebrate classes (mammals, fish, amphibians, reptiles and birds), and their life cycles exhibit alternation between vertebrates and invertebrates. Most species develop in arthropod vectors, which may belong to different orders and families, while amphibian and fish parasites are transmitted by insects and leeches. *T. evansi* is only transmitted mechanically. The species of this genus have several stages, which are present in blood and/or tissues in vertebrate and invertebrate hosts [1–4].

The genus *Trypanosoma* is monophyletic, with a common ancestor for all species found in mammals, birds, reptiles, amphibians and fish [3, 5–14]. In the most recent phylogenetic trees, three major clades were defined: *T. brucei*, *T. cruzi* and an aquatic clade (trypanosomes of fish and amphibians). Smaller clades have also been well established for other species: *T. lewisi*, *T. theileri*, *T. avium*/*T. corvi*, *T. grayi* and trypanosomes from lizards and snakes [4, 15–19]. Recently, new species from bats, rodents and marsupials were described in Australia and Africa [8, 20].

The order Perissodactyla comprises ungulate mammals of three different families: Equidae (horses, asses and zebras), Rhinocerotidae (rhinoceroses) and Tapiridae (tapirs) [21]. Only four species have been described in the Tapiridae family: two are present in South America (*Tapirus terrestris* and *Tapirus pinchaque*), one in Central America (*Tapirus bairdii*) and one in Asia (*Tapirus indicus*) [21]. In the Americas, *T. terrestris* is widely distributed in much of South America, including Colombia, Venezuela, Suriname, Guyana, French Guiana, Ecuador, Peru, Bolivia, Brazil, Paraguay and Argentina [21].

The tapir has important ecological roles in forming and maintaining biological diversity [22, 23]. However, the species is currently on the Red List of the International Union for Conservation of Nature (IUCN) as “Vulnerable to Extinction” because of the population decline that has occurred over the past thirty-three years (three generations), caused by illegal hunting, roadkill, loss and fragmentation of habitat and competition with livestock [24]. In Brazil, the lowland tapir is considered Endangered in the Atlantic Forest by the last Assessment of the State of Conservation of Ungulates and in the state of Espírito Santo, tapirs are also considered Endangered [25, 26]. Diseases are often mentioned as a serious threat to species conservation [27]. In captive and free-living tapir populations, pathogens such as bacteria, fungi, worms and ectoparasites have been diagnosed [28–31]. However, there is only a single report of *T. evansi* infecting tapirs (*Tapirus indicus*), in Indonesia [32], and since then there have been no studies on this mammalian species.

In the present study, we inferred phylogenetic relationships among the trypanosomes of *Tapirus terrestris* and other species based on SSU rDNA and gGAPDH gene sequences. Additionally, phylogenies and morphological data, including data from scanning and transmission electron microscopy, were used in describing a new species, *Trypanosoma terrestris* sp. nov., from *Tapirus terrestris* caught in areas of the Atlantic Rainforest of southeastern Brazil.

## Methods

### Study areas and tapirs caught

Specimens of free-living Lowland tapirs (*Tapirus terrestris*) were caught in a fragment of the Atlantic Rainforest in the state of Espírito Santo state. The Private Natural Heritage Reserve Recanto das Antas (Reserva Particular do Patrimônio Natural, in Portuguese, hereafter “RPPN”) which is the largest in Espírito Santo (2202 ha) is located in the municipality of Linhares (19° 5′ S, 39° 58′ W) and mostly consists of discontinuous secondary vegetation. The Corrego do Veado Biological Reserve (2382 ha) is located in the municipality of Pinheiros (40° 08′ 48″ S, 18° 20′ 33″ W) in the north of the state and was the last remainder of the Atlantic Rainforest in the region, since the natural vegetation has been replaced by agriculture and pasture. In addition, individuals that had been kept in captivity were used: these were from four different Brazilian states (São Paulo, Espírito Santo, Minas Gerais and Paraná).

The free-living tapirs were caught between March 2012 and July 2012 using box trapping and the tapirs that had been kept in captivity were sampled between January 2012 and March 2012. The tapirs were anesthetized as described by Mangini [33] and blood samples were collected by means of cephalic vein puncture.

### Isolation of trypanosomes in tapirs through culturing

To detect the presence of trypanosomes, the blood samples were inoculated into Vacutainer tubes containing a biphasic medium consisting of 15% sheep red blood cells as the solid phase (blood agar base), overlain by liquid LIT medium supplemented with 20% FBS [34, 35]. The culture was incubated at 28°C and grown in LIT medium for DNA preparation, and the isolates were cryopreserved in liquid nitrogen in the Brazilian Trypanosomatid Collection (Coleção Brasileira de Tripanossomatídeos, CBT), in the Department of Preventive Veterinary Medicine and Animal Health, Faculty of Veterinary Medicine, University of São Paulo, Brazil. Blood samples were fixed in ethanol (primary samples) for molecular detection.

### Growth behavior and morphological characterization

Epimastigotes and metacyclic trypomastigotes (1 × 10^6^ parasites) were used to infect monolayers of mammalian cells (Vero and J774) that had been cultivated in RPMI medium, with 10% FBS at 28°C, and insect cells (C6/36 and SF9) cultivated with L15 and SF9 II, respectively, with 10% FBS at 28°C. Additionally, BALB/c mice were inoculated (intraperitoneally) with cultured metacyclic forms (~10^7^/animal) of *T. terrestris* (CBT 46) obtained in stationary phase and were examined by means of MH three times per week from day 5 to day 30. After this period, blood samples were evaluated by means of culturing.

Smears were taken from cultures in LIT medium in the logarithmic and stationary phases, from the isolates CBT 41 and CBT 60 and from tapir blood, and were fixed in methanol and stained with Giemsa for light microscopy.

For scanning electron microscopy, trypanosomes from an axenic CBT 46 culture in LIT medium were fixed in Karnovsky (v/v), in 0.1 M cacodylate buffer (pH 7.4) for 2 h. After fixing, the parasites were set in 0.1% poly-L-lysine on coverslips and were dehydrated in an ascending ethanol series and in acetone (95%), and then in a critical point dryer (Balzers CPD 030). The processed material was then sputtered with gold (using Sputtrering Balzers SCD 050) and was observed using a HITACHI TM3000 digital scanning electron microscope.

For transmission electron microscopy, trypanosomes from CBT46 in the logarithmic phase were fixed in 2.5% glutaraldehyde fixative solution in 0.1 M sodium cacodylate buffer (pH 7.2) for 2 h. Two 15-min washes in cacodylate buffer followed this process. After washing, the material was post-fixed in 1% w/v osmium tetroxide in the same buffer and was contrasted with uranyl acetate for 12 h. Dehydration was carried out using graded acetone and embedding was performed using epon-araldite resin for 12 h at 60°C. Thin sections were stained with uranyl acetate and lead citrate [34] for 45 min and 10 min, respectively. Then, they were analyzed and photographed in a Philips CM 100 TEM, in the Biology Department of the Biosciences Institute, UNESP, Rio Claro campus, Rio Claro, SP, Brazil.

### Molecular and phylogenetic analysis

DNA was extracted from the trypanosome culture samples using the phenol-chloroform method, and primary samples (blood preserved in ethanol) were purified using the Wizard DNA Clean-Up System (Promega). The DNA samples were subjected to the conventional polymerase chain reaction (PCR) for the full length of SSU rDNA and gGAPDH genes, as previously described [18–20]. Trypanosome barcoding was used to detect the presence of trypanosomes in primary samples [20, 36, 37]. PCR products of the expected size were purified and sequenced in an automated sequencer (Applied Biosystems/PerkinElmer, model ABI Prism 310 Genetic, Foster City, California), in accordance with the manufacturer’s recommendations. All these sequences were retrieved from GenBank (accession numbers: SSU rDNA/gGAPDH): *Herpetomonas samuelpessoai* (U01016/AF047494), *H. megaseliae* (U01014/DQ092547), *H. muscarum* (L18872/DQ092548), *Phytomonas* sp. (AF016322/AF047496), *Leishmania major* (AF303938/AF047497), *L. tarentolae* (M84225/DQ092549), *Crithidia fasciculata* (Y00055/AF053739), *Leptomonas* sp. Nfm (AF153043/AF339451), *L. peterhoffi* (AF153039/AF322390), *Wallaceina brevicula* (AF153045/AF316620), *Trypanosoma rotatorium* (AJ009161/AJ620256), *T. mega* (AJ009157/AJ620253), *T. fallisi* (AF119806/AJ620254), *T. binneyi* (AJ132351/AJ620266), *T*. sp. K&A (AJ009167/AJ620252), *T. granulosum* (AJ620551/AJ620246),*T*. sp. CLAR (AJ620555/AJ620251), *T*. sp. Gecko (AJ620548/AJ620259), *T. varani* (AJ005279/AJ-620261), *T. cascavelli* (EU095837/FJ236511), *T. grayi* (AJ620546/AJ620258), *T*. sp 610 (EU596252/EU596256), *T*. sp 624 (EU596253/EU596257), *T*. sp 1092 (EU596254/EU596258), *T. vivax* (U22316/AF053744), *T. brucei rhodesiense* (AJ009142/AJ620284), *T. evansi* (AJ009154/AF053743), *T. simiae* (AJ009162/AJ620293), *T. congolense* (U22318/AJ620291), *T*. sp. AAT (AJ620557/AJ620264), *T. avium* rook (U39578/AJ620262), *T. avium* chaffinch (AJ009140/AJ620263), *T*. sp. D30 (AJ009165/AJ620279), *T. theileri* (AJ009164/AJ620282), *T. cyclops* (AJ131958/AJ620265), *T*. sp. TL.AQ.22 (AJ620574/AJ620280), *T*. sp. ABF (AJ620564/AJ620278), *T*. sp. H25 (AJ009168/AJ620276), *T. erneyi* (JN040987/JN040964), *T. erneyi* (JN040988/JN040965), *T. dionisii* (AJ009151/AJ620271), *T. cruzi marinkellei* (AJ009150/AJ620270), *T. cruzi* (AJ009147/X52898), *T. cruzi* (AJ009149/AJ620269), *T. rangeli* (AJ009160/AF053742), *T. rangeli* (*minasense*) (AJ012413/AJ620274), *T. vespertilionis* (AJ009166/AJ620283), *T. conorhini* (AJ012411/AJ620267), *T*. sp. F4 (AJ620547/AJ620260), *T. pestanai* (AJ009159/AJ620275), *T*. sp. AAP (AJ620558/AJ620277), *T. lewisi* (AJ009156/AJ620272), *T*. sp. R1 (AJ620568/AJ620281), *T. microti* (AJ009158/AJ620273), *T*. sp KG1 (AB281091/FJ649492), *T. gilletti* (GU966588/GU966587), *T. copemani* (GU966588/GU966585) and *T. copemani* (AJ620588/GU966586).

The sequences obtained were aligned with sequences that had previously been determined for other trypanosomatid species available in GenBank, using ClustalX [35], and were adjusted manually using GeneDoc [38]. The full-length SSU rDNA and gGAPDH sequences were concatenated with the 2898 characters obtained. This was used to construct a phylogenetic tree using maximum parsimony, as implemented in PAUP version 4.0b10 [39] with 500 bootstrap replicates, random stepwise addition starting trees (with random addition sequences) and TBR branch swapping. Bayesian analysis was performed using MrBayes v3.1.2 [40] with four independent Markov chain runs for 1,000,000 metropolis-coupled MCMC generations, sampling a tree every 100^th^ generation. The first 25% of the trees represented burn-in, and the remaining trees were used to calculate Bayesian posterior probability.

### Ethical approval

The animals were caught and manipulated in accordance with the recommendations of the Brazilian Institute for the Environment and Renewable Natural Resources Chico Mendes Institute for Biodiversity Conservation (IBAMA ICMBio) and approved by the Animal Research Committee of the School of Veterinary Medicine, University of São Paulo.

## Results

### Culturing of trypanosomes found in tapirs

Blood samples were collected from three free-living individuals that were caught in three different areas of the Atlantic Rainforest in the state of Espírito Santo: Corrego do Veado Biological Reserve in the municipality of Pinheiros; and Recanto das Antas Private Reserve in the municipality of Linhares; in the municipality of Marechal Floriano. Twenty-one individuals that were kept in captivity in several Brazilian states were also sampled. All the free-living tapirs were positive for trypanosome parasites in the cultured samples: this contrasted with the individuals kept in captivity, which were negative in blood cultures, except for one individual that was kept in captivity in Marechal Floriano and presented a positive blood culture. The prevalence values were 100% and 4.7%, among the free-living and captive Brazilian tapirs, respectively.

Blood samples (primary samples) from all the individuals of *Tapirus terrestris* that were caught were used for molecular screening with trypanosomatid barcoding. All the individuals sampled were negative, including the free-living tapirs with positive blood cultures.

### Growth, behavior and morphology

The three isolates were able to grow in LIT, RPMI and TC-100 media with 10% FBS. Monolayers of Vero, C6/36 and SF9 cells favored parasite growth, but it was not possible to verify invasion in insect and mammalian cells. In J774 cells (macrophage lineage), there was no growth of trypanosomes. In stationary culture, after 20 days post inoculation, large and slim flagellates with terminal end kinetoplast were predominant and resembling metacyclic trypomastigote culture forms. Additionally, metacyclic trypomastigote culture forms were inoculated into Balb/c mice and no infection was detected by means of microhematocrit or blood culturing.

**Figure 1:**
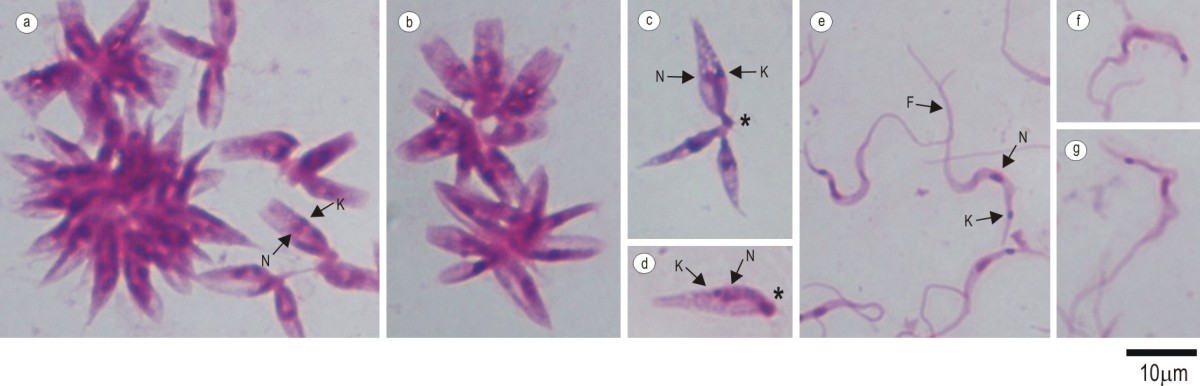
**Light microscopy of** ***Trypanosoma terrestris.*** Photomicrographs (Giemsa-stained) of LIT culture forms from *Trypanosoma terrestris* sp. nov. Epimastigote forms from logarithmic phase arranged in rosettes with kinetoplast and nucleus shown **(a-d)** without free flagellum **(c-d)** and flagellar sheath (*). Metacyclic trypomastigote forms from stationary phase **(e-g)**. N, nucleus; K, kinetoplast; F, flagellum.

Epimastigote forms do not present a free flagellum and motility was characterized by a large variability of movements and trajectories with alternating periods of translational movement, tumble, and shutdown of the parasites. Morphologically parasites were large and wide and exhibited a large rounded kinetoplast positioned near to the nucleus (Figure 1a-d). The epimastigote only grew in rosettes grouped in the anterior region (Figure 1a-c), and the darkly stained structure was similar to a flagellum sheath (Figure 1c, d). Metacyclic trypomastigote culture forms were represented by large trypanosomes with a long drawn out and pointed posterior end with the kinetoplast more distant from the posterior end (Figure 1e-g). The flagellum was almost the size of the trypomastigote body. *Trypanosoma terrestris* did not show amastigote or spheromastigote forms in axenic cultures or monolayer cells. No trypomastigote forms were detected in blood smears from positive tapirs.

**Figure 2:**
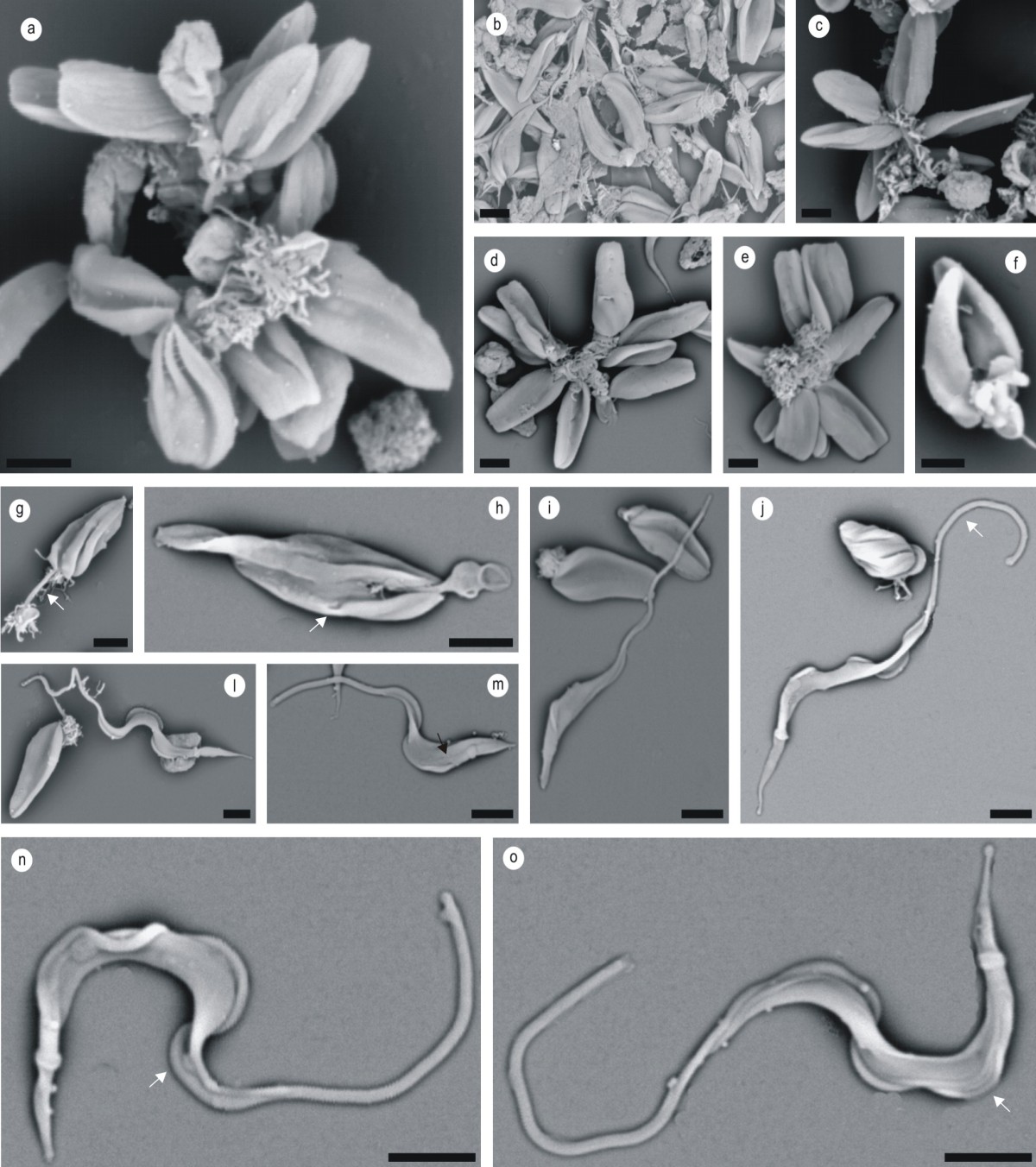
**Scanning electron microscopy on** ***T. terrestris*** **.** Epimastigote forms in rosettes **(a-f)** or separate **(f-h)**, twisted epimastigote **(g)** in metacyclogenesis **(i, j, m)** and flagellar sheath **(h)**. Cytosome indicated by arrows **(h, m)**. Metacyclic trypomastigote forms with long free flagellum **(j, n, o)** and undeveloped undulating membrane, indicated by arrows **(n, o)**. Scale bar 10 μm.

The scanning electron microscopy on cultured forms of *T. terrestris* displayed an interesting set of morphological characteristics that is uncommon among trypanosomes. Epimastigote forms of *T. terrestris* were organized in rosettes linked by a flagellar region, but no free flagellum was shown (Figure 2a-f). In the stationary phase and during metacyclogenesis, the epimastigote forms detached from the rosettes (Figure 2f-h) and became elongated (Figure 2i, l, m). During this process, a structure similar to a pocket was observed, sheltering the flagellum (Figure 2g, h). In metacyclic trypomastigotes, the flagellum emerged below the region of the kinetoplast and remained attached to the plasma membrane in the anterior region, with a long free segment and a very narrow undulating membrane (Figure 2j, n, o).

**Figure 3:**
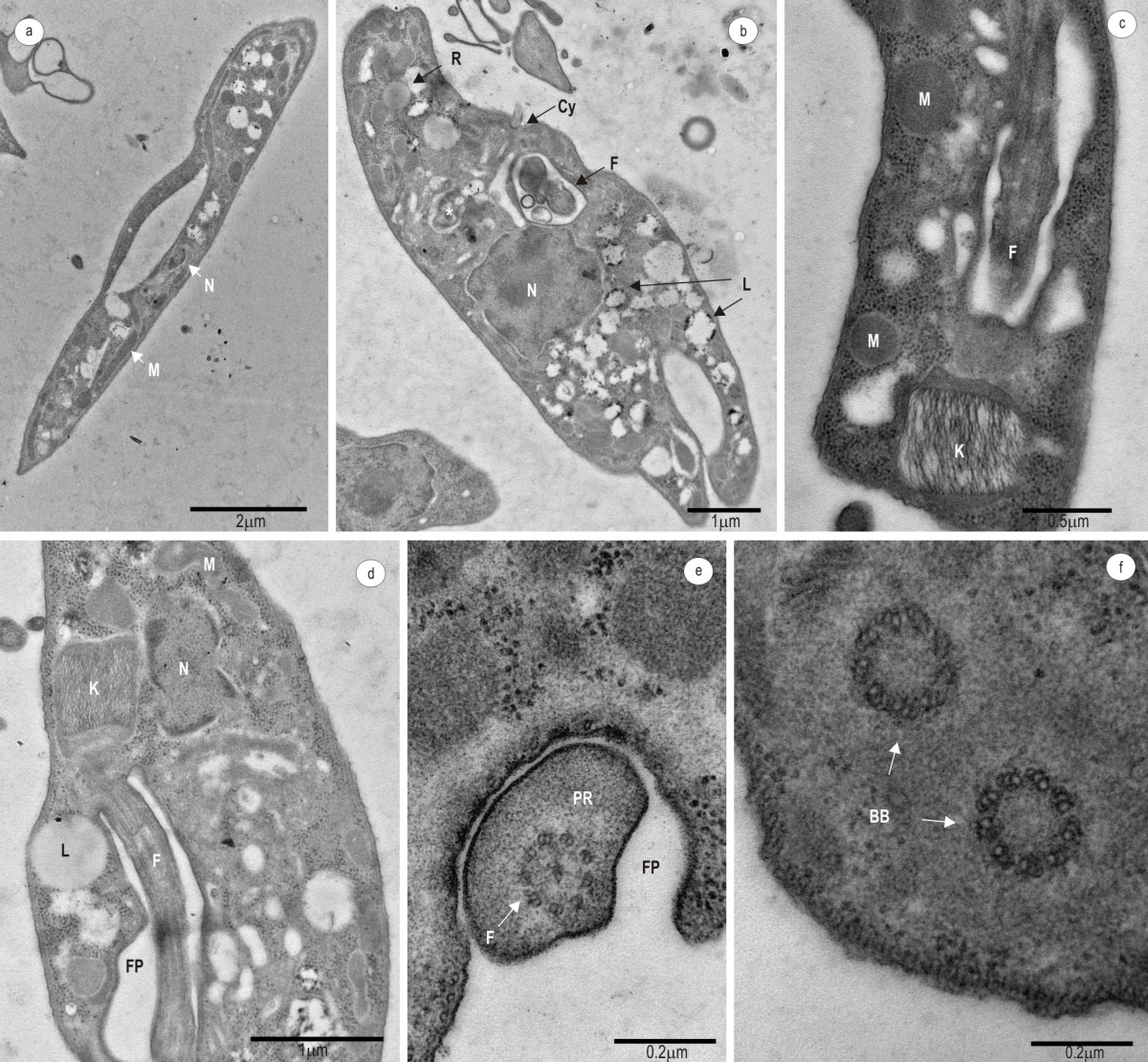
**Transmission electron microscopy on** ***T. terrestris*** **.** Logarithmic and stationary phase from epimastigote forms **(a, b, d, f)** and trypomastigote forms **(c, e)**. Ultrastructural organization, mitochondria, nucleus and kinetoplast common to *Trypanosoma* **(a-d)**. Large and loose kinetoplast **(c, d)**. Multiple lysosomes **(b, d)**, reservosomes **(b)**, cytosome **(b)**. Flagellum and paraxonemal structure **(c-e)**, well developed flagellar pocket **(d-e)** and basal bodies in a structure that shelters the flagellum in epimastigote forms **(f)**. Contractile vacuole (*). M, mitochondria; N, nucleus; K, kinetoplast; F, flagellum; R, reservosome; L, lysosome; Cy, cytosome; FP, flagellar pocket; PR, paraxial rod; BB, basal bodies.

Transmission electron microscopy identified ultrastructural organization common to other *Trypanosoma* species, such as a single mitochondria, nucleus and kinetoplast (Figure 3a-d). The trypanosomes have a large and loosely arranged kinetoplast (Figure 3c, d). Structural profiles compatible with a contractile vacuole can be observed near the flagellar pocket (Figure 3b). There were multiple lysosomes (Figure 3b, d), reservosomes (Figure 3b), a cytosome (Figure 3b), flagellum and paraxonemal structure (Figure 3c-e) and a well-developed flagellar pocket (Figure 3d-e). Basal bodies were observed in a structure that sheltered the flagellum in epimastigote forms (Figure 3f).

### Phylogenetic analysis

The SSU rDNA and gGAPDH sequences obtained for three *T. terrestris* isolates demonstrated low intraspecific divergence (~0.98%) and BLAST analysis showed that these isolates were closest to the trypanosomes of crocodiles (*T. grayi* and *T*. sp from Brazilian caimans).

**Figure 4:**
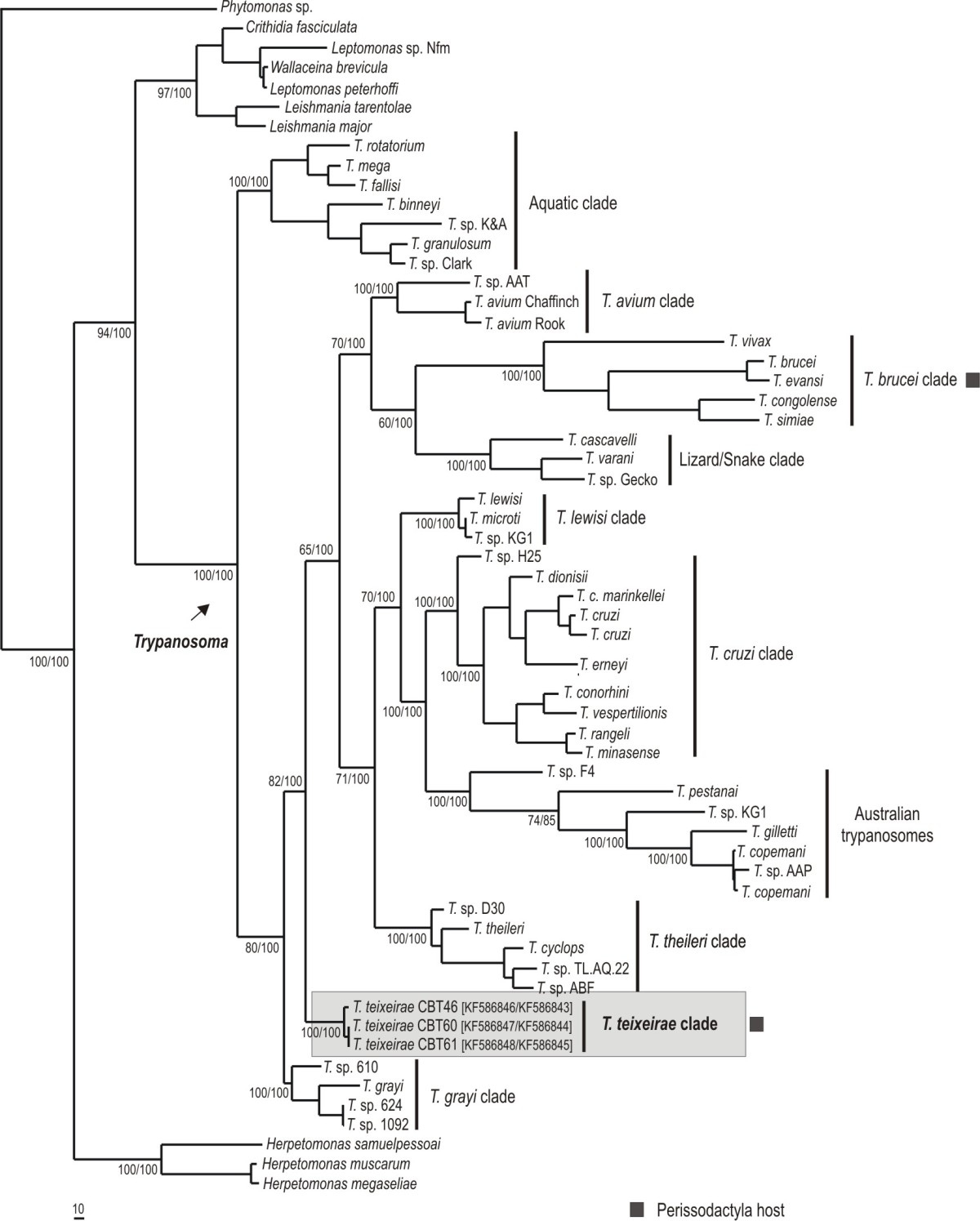
**Phylogenetic tree of a new trypanosome species,** ***Trypanosoma terrestris*** **, from Lowland tapir.** Phylogenetic tree based on concatenated SSU rDNA and gGAPDH gene sequences of 51 trypanosome isolates using non-trypanosome trypanosomatids as outgroup (2898 characters and 875 parsimony-informative sites), which was used in parsimony and Bayesian methods. Numbers at nodes are support values for the major branches (bootstrap/posterior probability; 500 replicates). *T. terrestris* clades are bold and in gray box.

Phylogeny based on concatenated SSU rDNA and gGAPDH genes and inferred through parsimony and Bayesian analyses generated trees with identical topologies and corroborated the phylogenetic relationships of other species and trypanosome clades, with well-supported clades for trypanosomes. In all the inferred trees, the *T. terrestris* isolates were grouped together and were distinct from other trypanosome species and were not grouped with any known clades (Figure 4). The isolates from *Tapirus terrestris* (*T. terrestris*) in Brazil formed a new clade with high divergence from the major known clades, of between 9.67% and 28.89%, with the clades for *T. grayi* and Australian trypanosome species (*T. copemani*, *T. gilletti*, *T*. sp ticks and others), respectively. *T. terrestris* isolates also presented divergences of 11.94%, 13.15%, 13.41%, 13.88%, 14.55%, 14.38%, 16.09% and 19.88% with the clades for lizards/snakes, *T. cruzi*, *T. avium*, *T. lewisi*, aquatic species, *T. brucei* and *T. theileri*, respectively. The high divergences between *T. terrestris* and the other species or major clades of the genus *Trypanosoma* and the tree topology demonstrate that this new species forms a new clade in a monophyletic group of trypanosomes (Figure 4). In accordance with these results, we have designated a new species and new clade named *Trypanosoma terrestris*.

### Taxonomic summary

Phylum Euglenozoa Cavalier-Smith, 1981

Class Kinetoplastea Konigberg, 1963

Order Trypanosomatidae (Kent 1880) Holande, 1952

Family Trypanosomatidae Doflein, 1901

Genus *Trypanosoma* Gruby, 1843

### *Trypanosoma terrestris* Marcili n. sp.

#### Morphology

Epimastigote forms were large with a straight posterior end. These forms did not have a free flagellum and motility was characterized by a large variability of movements and trajectories with alternating periods of translational movement, tumble, and shutdown of the parasite. The epimastigote only grew in rosettes grouped anteriorly, and exhibited a rounded and large kinetoplast positioned near to the nucleus. Metacyclic trypomastigote culture forms were represented by large trypanosomes with a long drawn out and pointed posterior end. The undulating membrane in metacyclic trypomastigote forms were slightly developed and its outer margin forming only a few shallow convolutions. The flagellum extends beyond the anterior end of the body as a free long flagellum and was almost the size of the trypomastigote body. The kinetoplast was more distant from the posterior end. Data on blood trypomastigote were not available.

#### Type host

Mammalia, Perissodactyla, Tapiridae, *Tapirus terrestris*.

#### Vector

Unknown.

#### Type locality

The specimens were caught in the Atlantic Rainforest biome, Córrego do Veado Biological Reserve (40° 08′ 48″S, 18° 20′ 33″W), municipality of Pinheiros, state of Espírito Santo, southeastern Brazil.

#### Type material

Hapantotype, culture of the isolate CBT 60; paratypes, cultures CBT 46 and CBT 61. The cultures have been deposited in the Brazilian Trypanosomatid Collection, at the Faculty of Veterinary Medicine, University of São Paulo, Brazil.

#### Gene sequences

The SSU rDNA (GenBank:KF586847/KF586846/KF586848) and gGAPDH (GenBank:KF586844/KF586843/KF586845) sequences from *Trypanosoma terrestris* (hapantotype CBT 60 and paratypes CBT 46 and CBT 61, respectively) have been deposited.

#### Etymology

The name has been given according the host name Tapirus terrestris.

#### Comments

The hosts of paratypes CBT 46 and 61 were caught in the Atlantic Rainforest biome, in the municipalities of Linhares (19°5′S, 39°58′W) and Marechal Floriano (20° 24′ 46″S, 40° 40′ 59″W), respectively, in the state of Espírito Santo, southeastern Brazil.

#### General

In accordance with section 8.5 of the ICZN’s International Code of Zoological Nomenclature, details of the new species have been submitted to ZooBank with the life science identifier (LSID) zoobank.org:pub: 3FF1C1D7-44AC-4D70-9D3B-41426B8FC2E7.

## Discussion

In this study, morphological analysis based on light microscopy, transmission and scanning electron microscopy and phylogenetic positioning made it possible to describe a new species, *Trypanosoma terrestris* sp. n. from *Tapirus terrestris*. This is the first description of a trypanosome in *Tapirus terrestris* (Lowland tapir), the largest mammal in Brazil [25]. It has singular morphology with large and wide epimastigote forms without a free flagellum and active movement and flagellar beating. The concatenated phylogeny (SSU rDNA and gGAPDH genes) showed high divergences in comparison with other trypanosome clades, and the *T. terrestris* isolates were positioned in a new clade named *T. terrestris* clade.

The morphological characteristics shown by light microscopy and electron microscopy are mostly similar to those of other species of trypanosomes, with the exception of the absence of free flagella in epimastigotes. This characteristic has only previously been described in a trypanosome infecting kangaroos in Australia, which also has no free flagellum in epimastigotes, but has only been described in light microscopy [41]. These authors described this structure as hemidesmosomes with adhesion in the gut of the invertebrate hosts or vectors, but this was only seen using light microscopy [41].

Traditional parameters for the description of *Trypanosoma* species include the morphological description of blood forms in vertebrate hosts. Unfortunately, blood smears for positive tapirs in hemoculture were negative for trypomastigote forms, probably reflecting low parasitemia and similar to other recent trypanosomes descriptions [20, 42]. However, metacyclic trypomastigotes were obtained from culture media and described.

*T. terrestris* was able to grow in culture media and cells with low nutritional requirements, but was not capable of infecting BALB/c mice. This suggests that the vertebrate host is specific. However, studies on experimental infection in other mammals of the order Perissodactyla, such as horses, are still needed.

The phylogenies based on whole SSU rDNA and gGAPDH alone or on concatenated analysis have been used to position new trypanosomatid species and genera [20, 42–44]. The divergence between *T. terrestris* isolates and those of other species ranged from 9.67% to 28.89%, thus making it difficult to accurately position the new species in the *Trypanosoma* clades, and hence a new clade was proposed for classifying *T. terrestris*.

The vectors for the trypanosomes of the Lowland tapir remain unknown. These hosts can be infested by *Amblyomma* sp. ticks [45], Hipoboscidae dipterans [46], leeches [47] and probably culicids and tabanids. Therefore, these are possible vectors of *T. terrestris. Trypanosoma* species that infect ungulates, such as *T. theileri, T. vivax* and *T. evansi* are transmitted by many hematophagous dipterans [48, 49].

Among the tapirs in captivity that were sampled, only one was positive, whereas all of the free-living individuals were positive. This single animal in captivity that was infected with *T. terrestris* had escaped from captivity and it had remained free-living in a sylvatic environment for six months before collection of blood samples. Animals kept in captivity are regularly treated against ectoparasites, unlike free-living tapirs, which are constantly exposed to possible vectors.

The order Perissodactyla or odd-toed ungulates is a very old group of mammals that appeared at the start of the Eocene, approximately 55 Mya [50, 51] and became widely distributed in North America, Latin America, Europe and Asia, while remaining absent from Australia and Antarctica [52, 53]. Divergence within Perissodactyla occurred on the Laurasian continent around 56 million years ago, and this scenario is supported by paleontological and molecular data [54–57].

The Tapiridae family is composed of a single genus, i.e. *Tapirus*. The oldest fossil record of this family has been dated as Oligocene, in Europe (from 33 to 37 Mya) and records have frequently been found in Europe, North America and Asia [58]. The earliest record of *Tapirus* was in the Oligocene and fossil remains are found up to the Pleistocene [53]. In North America, records relating to *Tapirus* indicate that they were present in the Middle Miocene [58], while in Asia the records indicate that *Tapirus* has existed since the lower Miocene. About twenty different species of *Tapirus* have been recognized in Europe, South America, North America and Asia [59].

The high degree of divergence between *T. terrestris* and other *Trypanosoma* species suggests that divergence occurred early on, along with their hosts. New studies need to be conducted on other *Tapirus* and Perissodactyla species in order to confirm the coevolutionary patterns.

The tapir populations have become increasingly isolated throughout their geographical distribution, thus the population decline or local extinction of these animals may trigger a series of negative effects on ecosystems [60]. It may destabilize some ecological processes such as seed dispersal and seed predation [60–62], and could have an impact on plant recruitment patterns leading to a decline in plant diversity, thus compromising the integrity and biodiversity of the ecosystem over the long term [60, 63].

The Atlantic Rainforest is one of the most threatened biomes on the planet. At the time of the discovery of Brazil, the Atlantic Rainforest covered 1.4 million km^2^ and extended from Rio Grande do Sul to Rio Grande do Norte [64]. Today, the Atlantic Rainforest has been reduced to about 7% of its original length, and over 80% of the fragments are less than 50 hectares [65]. Despite the removal of this biome, survival and persistence of tapir populations may be favored by the ability of these animals to move into habitats that are available and appropriate [66, 67].

Diseases are often referred to as serious threats to species conservation [25]. The habitat of lowland tapirs in increasingly threatened and fragmented due to the increase in human population densities and demand for livestock products. These changes have increased the exchanges of pathogens between wild and domestic animals [28]. However, little known about the health and general factors affecting the lowland tapir in the natural environment [33], and most of the information about diseases in tapirs comes from captive tapirs [68]. The infections that have been described in lowland tapirs include *Streptococcus*, *Klebsiella*, *corynebacteria*, *Actinomyces*, *Fusobacterium*, *Salmonella*, *Campylobacter*, *Escherichia coli*, *Staphylococcus aureus*, *Leptospira* spp., *Ballantidium* spp., *Clostridium* spp., *Mycrosporum gypseum*, *Mycrosporum kennels*, *Trichophytuon tonsurans*, herpesvirus, *Giardia* spp., mouth disease, babesiosis, *Tapironema coronatum*, *Sarcoptes* hut, *Calpe eustrigata*, *Fasciola hepatica* and schistosomiasis [68–73]. There is a single report of *T. evansi* infection in *T. indicus*[32].

Further studies should be conducted to evaluate the pathogenicity of *T. terrestris* for the lowland tapir. These may help in defining whether its population decline means that it is endangered and, furthermore, may help in assessing the presence and pathogenicity of *T. terrestris* for domestic animals.

## Conclusions

Morphological and phylogenetic evidences of trypanosomes of *Tapirus terrestris* isolated in this study enable the descriptions of a new species and *Trypanosoma* clade named *Trypanosoma terrestris*. This was the first study to investigate, isolate and phylogenetically position trypanosomes in lowland tapirs.

## Authors’ original submitted files for images

Below are the links to the authors’ original submitted files for images. Authors’ original file for figure 1 Authors’ original file for figure 2 Authors’ original file for figure 3 Authors’ original file for figure 4
