# Supplementary figures and images for: Morphological and molecular characterization and phylogenetic relationships of a new species of trypanosome in Tapirus terrestris (lowland tapir), Trypanosoma terrestris sp. nov., from Atlantic Rainforest of southeastern Brazil
Source: Parasit Vectors. 2013 Dec 11;6:349. doi: 10.1186/1756-3305-6-349 (PMC3878878; doi:10.1186/1756-3305-6-349)

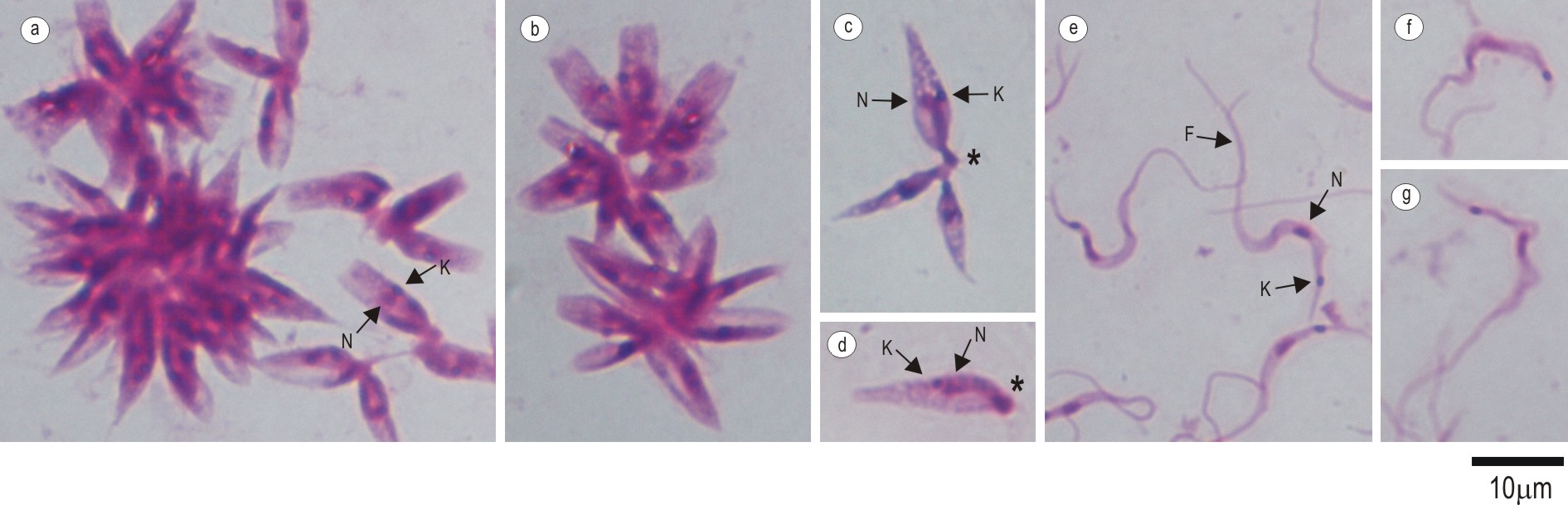

Supplement: Supplementary file 1 — Authors’ original file for figure 1 [file 13071_2013_1136_MOESM1_ESM.jpeg]

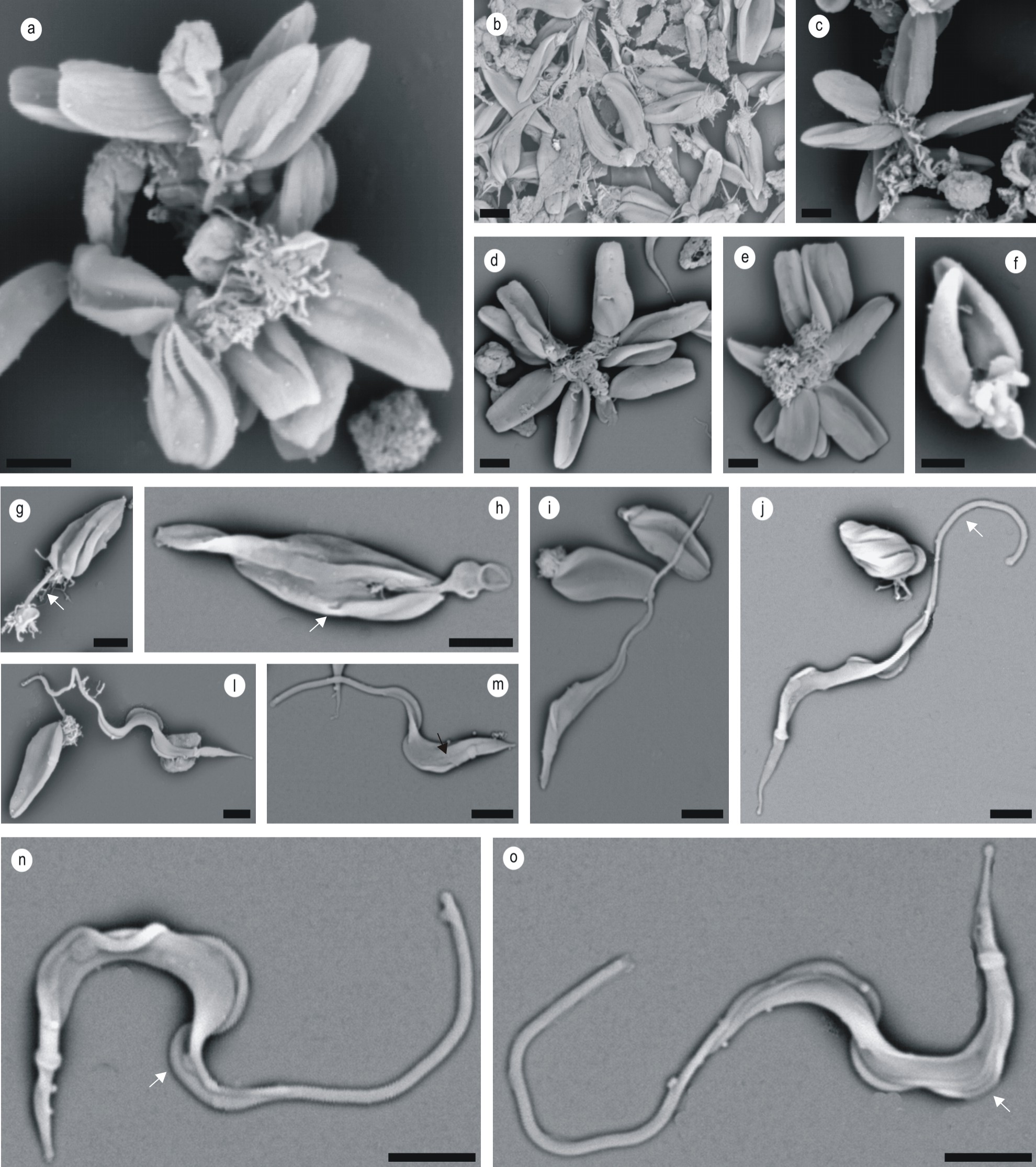

Supplement: Supplementary file 2 — Authors’ original file for figure 2 [file 13071_2013_1136_MOESM2_ESM.jpeg]

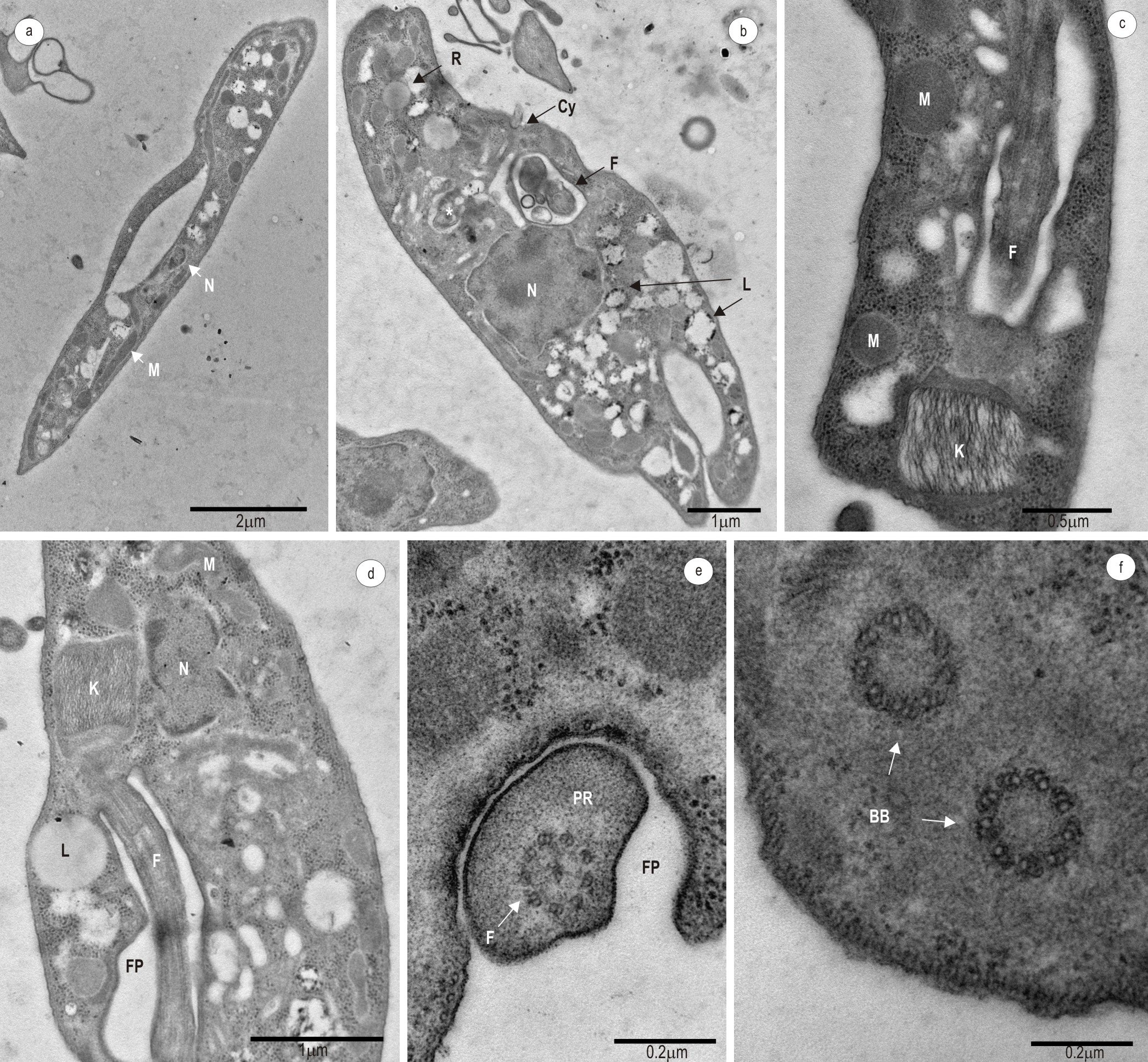

Supplement: Supplementary file 3 — Authors’ original file for figure 3 [file 13071_2013_1136_MOESM3_ESM.jpeg]

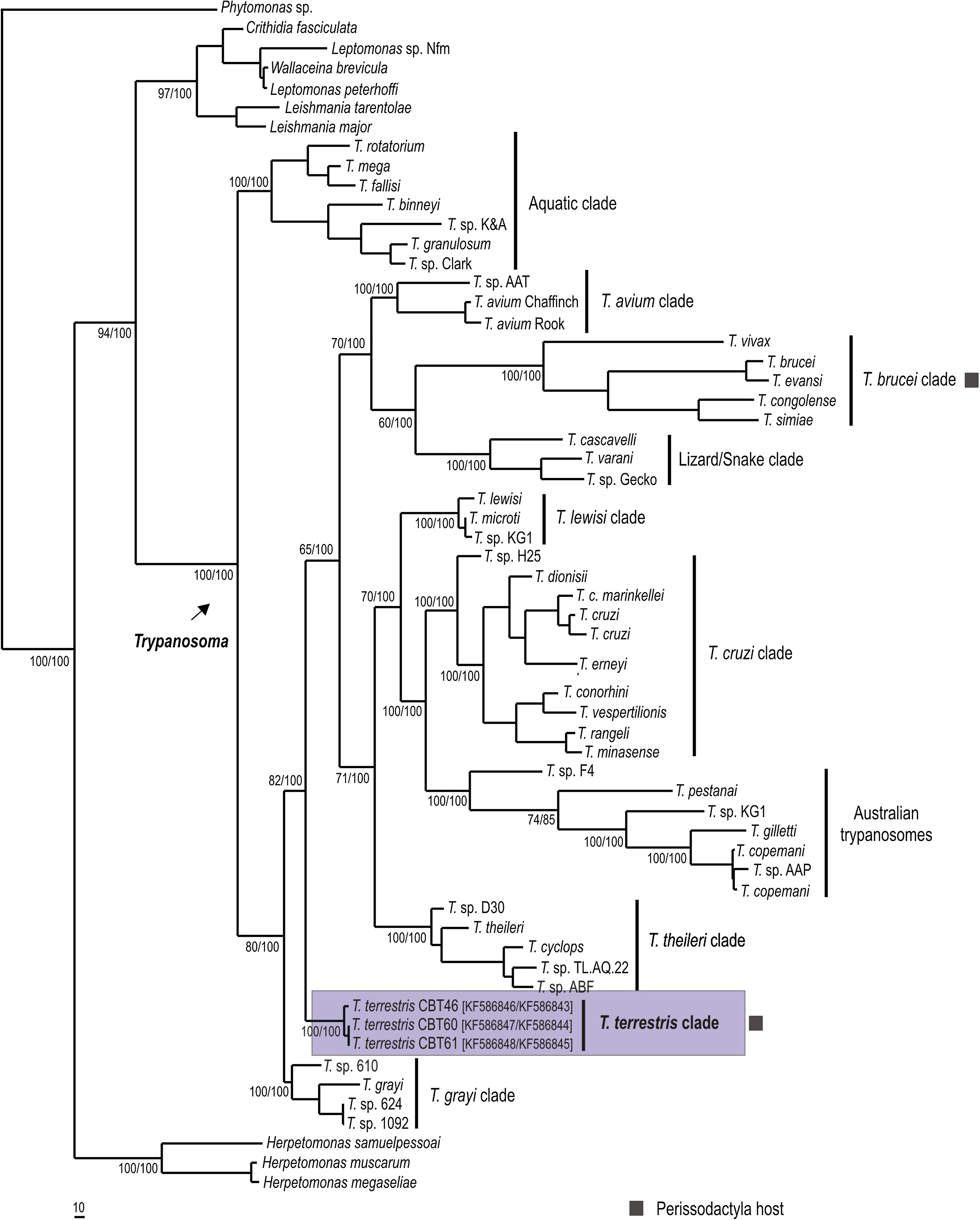

Supplement: Supplementary file 4 — Authors’ original file for figure 4 [file 13071_2013_1136_MOESM4_ESM.tiff]
